# Alexithymia Is Associated with Reduced Quality of Life and Increased Caregiver Burden in Parkinson’s Disease

**DOI:** 10.3390/brainsci10060401

**Published:** 2020-06-24

**Authors:** Martin Klietz, Theresa Schnur, Simon C. Drexel, Florian Lange, Lejla Paracka, Meret K. Huber, Dirk Dressler, Günter U. Höglinger, Florian Wegner

**Affiliations:** 1 Department of Neurology, Hannover Medical School, Carl-Neuberg-Straße 1, 30625 Hannover, Germany; Theresa.Schnur@stud.mh-hannover.de (T.S.); Simon.C.Drexel@stud.mh-hannover.de (S.C.D.); Paracka.Lejla@mh-hannover.de (L.P.); Huber.Meret@mh-hannover.de (M.K.H.); Dressler.Dirk@mh-hannover.de (D.D.); Hoeglinger.Guenter@mh-hannover.de (G.U.H.); Wegner.Florian@mh-hannover.de (F.W.); 2 Behavioral Engineering Research Group, KU Leuven, Naamsestraat 69, 3000 Leuven, Belgium; florian.lange@kuleuven.be

**Keywords:** Parkinson’s disease, caregiver burden, alexithymia, health-related quality of life

## Abstract

Parkinson’s disease (PD) is the second most frequent neurodegenerative disease of people who are beyond 50 years of age. People with PD (PwP) suffer from a large variety of motor and non-motor symptoms resulting in reduced health-related quality of life (HR-QoL). In the last two decades, alexithymia was identified as an additional non-motor symptom in PD. Alexithymia is defined as a cognitive affective disturbance resulting in difficulty to identify and distinguish feelings from bodily sensations of emotional arousal. In PD, the frequency of patients suffering of alexithymia is increased compared to healthy controls. The aim of the present study was to determine the relationship of alexithymia to HR-QoL of the PwP and caregiver burden of the corresponding caregiver. This cross-sectional questionnaire-based study used disease specific questionnaires for HR-QoL and caregiver burden. In total 119 PwP and their corresponding caregivers were included in the study. HR-QoL of the PwP correlated significantly with alexithymia (*p* < 0.001), especially the sub-components “identifying feelings” (*p* < 0.001) and “difficulties describing feelings” (*p* = 0.001). Caregiver burden also correlated significantly with PwP alexithymia (*p* < 0.001). However, caregiver burden was associated with sub-components “identifying feelings” (*p* < 0.008) and “external oriented thinking” (*p* < 0.004). These data support the importance of alexithymia as a non-motor symptom in PD.

## 1. Introduction

Parkinson’s disease (PD) is a progressive neurodegenerative disease mainly affecting people beyond 50 years of age. In Germany, the prevalence of PD is estimated to be 0.5% of the population [1]. People with PD (PwP) suffer from classical motor symptoms like rigidity, tremors, bradykinesia, and postural instability [2,3]. These symptoms result from degeneration of the nigro-striatal dopaminergic projection [4,5]. However, PwP also suffer from neuropsychiatric symptoms like depression, anxiety, apathy, and impulse-control disorders [6,7,8,9]. Some neuropsychiatric symptoms may arise during the degeneration of the meso-limbic dopaminergic projection and secondary maladaptive changes [10,11,12,13,14].

With regard to these symptoms, PD patients display deficits in recognition and definition of emotions, also called alexithymia [15]. Taylor et al. described alexithymia as a cognitive affective disturbance defined as difficulty to identify and distinguish feelings from bodily sensations of emotional arousal [16]. Therefore, discrimination and expression of one’s own emotions and the interpretation of other peoples’ emotions might be impaired. Alexithymia is a multidimensional psychological construct based on the following four distinct elements: (1) difficulties in identifying and describing feelings; (2) difficulty in distinguishing between feelings and bodily sensations related to emotional activation; (3) restrained and limited imaginative processes, adopting the guise of an impoverished fantasy; and (4) a cognitive style oriented toward the outside [16]. People presenting with the personality trait of alexithymia knew little about their internal emotional states and were often unable in effectively relating them to previously experienced situations, memories, and fantasies [16,17].

These characteristics are widely distributed, including a limited recognition of emotional faces and voices [18,19,20,21]. In PD, about 18–23% of the patients meet the diagnostic criteria of alexithymia, which is twice as high as in the general population [20,22]. Alexithymia is closely related to depressed mood; nevertheless, it seems to be an independent symptom [23]. Importantly, alexithymia is associated with lower satisfaction in life, and affected people are more likely to commit suicide [24,25]. Until now, the impact of alexithymia on quality of life of PwP is still poorly understood, and the implications for the corresponding caregivers are missing. Therapeutic interventions for alexithymia in PD patients are rare—only one study reported beneficial effects from deep-brain stimulation of the subthalamic nucleus [26].

The aim of the present study is use disease-specific questionnaires to investigate the association of alexithymia in PwP with health-related quality of life (HR-QoL) and the burden on the corresponding caregivers.

## 2. Materials and Methods

### 2.1. Participants

This study was approved by the local Ethics Committee of Hannover Medical School (No. 3178-2016, Amendment in 2018). All patients and their caregivers gave their written informed consent to participate in the study. PwP and their caregivers were invited via the German PD association and the Hilde–Ulrichs Foundation. Local patients from the neurological wards and outpatient movement disorder clinic were included in this cross-sectional questionnaire-based study. Our sample included 119 PwP and related primary caregivers. Data from this patient and caregiver cohort unrelated to the current research question have already been reported elsewhere [27,28].

Inclusion criteria for PD patients were defined as neurologically determined Parkinson’s disease according to the MDS diagnostic criteria for PD with a duration of at least one year after onset of motor symptoms [29]. Patients suffering from atypical Parkinsonism were excluded from this study. Only the primary caregivers of the PwP were included. Professional caregivers were excluded from our study due to less emotional relation to the patient and different coping strategies. While professional caregivers were trained to do so, informal caregivers had mostly no specific training in caregiving or to develop coping strategies.

### 2.2. Measures

Caregivers and PwP were first asked to provide general background information (e.g., patients’ disease duration, daily amount of time caregivers dedicated to giving care to the patient, age, education, etc.).

PwP then completed the Movement Disorders Society unified Parkinson’s disease rating scale (MDS-UPDRS) part II to evaluate their impairment of daily living [30,31]. MDS-UPDRS is the most established scale for measurement of PD symptoms. It consists of four parts. Part I includes non-motor symptoms and their impairments of daily living. Part II assesses motor-impairments of daily living. In part III, a neurological scoring of the PD motor symptoms is performed, and in part IV, treatment complications are documented and scored. Importantly, some items of part I and whole part II can be scored by the PD patient possibly with assistance of the caregiver. MDS UPDRS part II consists of 13 items on a five-point Likert scale from 0 (no symptoms) to 4 (severe symptoms), with a maximum of 52 points indicating the worst extent of symptoms. The Hoehn and Yahr stage is one of the oldest disease stages for PD motor impairments. Stage I represents symptoms in only one body hemisphere and is the mildest amount of PD motor symptoms. Stage III is defined by gait and balance problems. Stage V is a wheelchair-bound or bedridden patient. Its assessment is included in the MDS-UPDRS.

To assess health-related quality of life (HR-QoL), PwP were requested to answer the Parkinson’s disease questionnaire 8 (PDQ-8) [32]. PDQ-8 is an excellent validated and established measure of HR-QoL in PwP. It assesses eight questions in eight domains. Items were on a five-point Linkert scale from 0 (best HR-QoL) to 4 (worst HR-QoL). Results are displayed as reduction in HR-QoL. Higher scores indicate worse quality of life of the PD patient. Because the PDQ-8 is only constructed and validated for PD patients, the HR-QoL of the caregiver could not be assessed by the PDQ-8. As done in other studies, caregivers of PwP with possible cognitive impairment were asked to assist patients in completing the study questionnaires to ensure correct results and avoid anosognosia [6,28,33]. Disease specific burden of the caregivers was assessed by the newly developed and validated German version of the Parkinson’s disease caregiver burden questionnaire (PDCB) [28,33,34].

Caregiver and patient depressive mood was assessed by Beck’s depression inventory (BDI) [35]. Caregivers’ HR-QoL was measured by the generic health-related quality of life questionnaire short form 36 health survey (SF-36) [36]. The SF-36 measures HR-QoL within eight dimensions. Hence, all subscale scores were highly correlated with each other. We calculated an average score across the eight scales as done in previous work of our group [27,33,36]. Further, we used the physical and mental component score of the SF-36. In general, all scores were transformed to a 0 (worst HR-QoL) to 100 (best HR-QoL) scale.

Alexithymia of PwP and caregivers was measured by the Toronto Alexithymia Scale with 26 items (TAS-26). Participants responded to each item on a five-point Likert scale. Sum scores were divided by the number of completed items to obtain average scores for the total scale and three facets/subscales assessing different domains of alexithymia (F1: difficulties identifying feelings, F2: difficulties describing feelings and F3: external oriented thinking). Participants with a converted total score of >3 were considered alexithymic according to the manual [16,37].

### 2.3. Statistical Analyses

Linear regression analyses were used to examine potential predictors of patients’ HR-QoL (PDQ-8) and caregiver burden (PDCB). To correct for multiple tests, the level of significance was set to α = 0.05/*n* (*n* = number of analyzed predictors). To test whether any predictors explained unique variances in our outcomes, we included significant predictors in a multiple regression model for patients’ HR-QoL and caregiver burden. Further, we first included TAS-26 total score in the multiple regression analysis when the total score showed a significant correlation, we replaced the total score by significant facets (e.g., TAS F1, F2, and/or F3) from the linear regression analysis. For the statistical analyses, SPSS 25.0 (IBM, Armonk, NY, USA) was used.

## 3. Results

### 3.1. Patient and Caregiver Characteristics

Table 1 summarizes demographic and clinical characteristics of the participating PwP and their primary caregivers. PwP were in mean in moderate to advanced stages of PD as characterized by Hoehn and Yahr stage 2.9 ± 1.1 and disease duration of 12.0 ± 7.6 years (mean ± SD). MDS-UPDRS II was 12.0 ± 7.6, and depressive symptoms resulted in a BDI score of 12.6 ± 8.1 indicating mildly depressed mood. Caregivers reported mild to moderate burden, scoring 37.1 ± 27.9 on the PDCB scale, and spent 5.8 ± 6.4 h per day caregiving. Depressive symptoms of the caregivers were lower than the reported BDI of the PwP (9.8 ± 7.3 vs. 12.6 ± 8.1). Interestingly, 36 PwP (33.2%) and 13 caregivers (11.9%) of our study sample were considered as alexithymic by the TAS-26 cut-off value. By comparing PwP considered as alexithymic to non-alexithymic PwP, significant differences in patients’ MDS-UPDRS II, PDQ-8, and BDI could be found (Table 2). All subscales of the TAS-26 were significantly different between both groups.

### 3.2. Factors Influencing HR-QoL of the PwP

First, linear regression analyses were performed to test whether variance of health-related quality of life (PDQ-8) of PwP could be explained by alexithymia of the patient measured by TAS-26. Disease duration, MDS-UPDRS II, BDI, TAS-26 total, TAS-26 subdomain F1 “identifying feelings” and subdomain F2 “difficulties describing feelings” correlated significantly with PwP quality of life (after correction for multiple comparisons). Interestingly, age and TAS-26 subdomain F3 “external oriented thinking” did not show a significant correlation with PwP quality of life. All correlations are presented in Table 3. For further testing for confounders and co-correlations all significant variables were included in a multiple regression analysis (Table 4). In this model MDS-UPDRS II, BDI and TAS-26 showed a robust significant correlation with PwP health-related quality of life (not shown). After that, we replaced TAS total score by the significant subdomains TAS F1 and TAS F2, by this we found MDS-UPDRS II, BDI, and TAS-F1 to be significantly correlated with HR-QoL of the PD patient (Table 4).

### 3.3. Factors Influencing Caregiver Burden in PD

PwP and caregiver factors were regressed to PDCB score of the caregiver. In the linear regression analysis PDQ-8, MDS-UPDRS II, PwP BDI, PwP TAS-26, PwP TAS-26 F1, PwP TAS-26 F2, caregiving hours per day, caregiver SF-36, caregiver BDI, and caregiver TAS-26 were significantly related to caregiver burden after controlling for multiple testing (Table 5). However, caregiver TAS-26 F1 and caregiver TAS-26 F3 were significantly associated with caregiver burden (both *p* < 0.05). To correct for confounder and co-correlations, all significant patient variables were included in a multiple regression analysis. In this analysis, only MDS-UPDRS II reached statistical significance (Table 6). Hence, because TAS-26 total was not significant in the multiple regression analysis, we did not include TAS subdomains in the analysis.

## 4. Discussion

In this cross-sectional questionnaire-based study we were able to investigate a cohort of PwP and their primary caregivers. PwP with alexithymia were found to report significantly lower quality of life. This relationship was especially pronounced for the alexithymia domains “identifying feelings” and “describing feelings.” Notably, PwPs’ difficulties in identifying feelings accounted for unique variance in health-related quality of life above and beyond disease duration, motor-symptom-related impairment, and depression. Alexithymia in the domains “identifying feelings” and “describing feelings” was also found to relate to increased caregiver burden. However, these facets of alexithymia did not reach significance in the multiple regression analysis.

Quality of life in PD is reduced in all disease stages. Contributing factors are motor and non-motor symptoms [6,9,28,38,39,40]. In the last decade, the influence of non-motor symptoms was extensively studied [41]. However, even during the last few years, several neglected non-motor symptoms have emerged or found new spotlights [26,40,42]. Our results support the role of alexithymia as a non-motor symptom contributing to impaired quality of life in PD. About one third of the PwP in our study were alexithymic. This is a larger proportion than described in previous studies with a prevalence of about 20% [20,26,43]. Reasons for the higher proportion of alexithymic PwP might be the more advanced disease stages in our group compared to other studies. The prevalence of alexithymia in PD caregivers was about 10% in our study and is described as similarly high in other studies investigating a non-PD group, which underlines the validity of the TAS-26 in detection of alexithymia. Further, the use of a different version of the Toronto Alexithymia Scale (26 vs. 20 items) might be a reason for different proportions of alexithymic PwP, since these two versions of the scale (e.g., TAS-20 and TAS-26) display slightly variations in their psychometric properties [44,45]. In the recent literature concerning alexithymia and Parkinson’s disease TAS-20 has mainly been used. The initial longer version TAS-26 has some limitations concerning the item structure and psychometric properties. Also, facet 4—“reduced daydreaming”—does not correlate with the other items. However, with the modification of the TAS-26 in the German translation, these limitations have been overcome [37]. Because of this, we decided to use the longer TAS-26 to avoid a loss of information. Also, we only analyzed facets 1–3, as the fourth item is no longer included in the new TAS-26. Data from Kupfer et al., demonstrated that the German version of the TAS-26 displays superior psychometrics compared to the German version of the TAS-20 [37]. Further, our data show a highly significant correlation with HR-QoL and a profound discrimination of alexithymic PD patients with worse HR-QoL from patients without alexithymia and better HR-QoL. In our opinion, both scales can be used in future studies. However, we also observe that most studies in PD used the TAS-20 and, therefore, the comparability of the data would be improved by using TAS-20 in future studies.

In our cohort, mean disease duration was 12 years. By comparison with non-alexithymic patients, we found that alexithymic patients were more severely affected by PD motor restrictions in activities of daily living (measured by the MDS-UPDRS II) displaying more depressive mood and quality of life restrictions. Additional, alexithymia correlated significantly with PwP HR-QoL, which was also found in other studies [26].

In patients with dystonia, a significant correlation of alexithymia and HR-QoL was recently shown by our group [46]. There are several studies in various diseases connecting the extent of alexithymia with decreased HR-QoL [47,48,49,50]. By taking this available evidence together, it seems that HR-QoL restrictions in patients with alexithymia might not be PD specific. Alexithymia seems to generally reduce HR-QoL in people with this personality trait [47]. This factor might be explainable because these alexithymic people were not able to describe their feelings and to understand the feelings of others. However, the mechanisms of reduced HR-QoL in alexithymia have not been fully understood yet. Despite the notion that HR-QoL restrictions due to alexithymia seems to be not specific for PD, as the number of people who could be classified as alexithymic might be elevated in PD [20]. The proportion of PwP characterized as alexithymia is about two times higher than in other diseases, emphasizing the impact of this phenomenon in PwP and their caregivers [20,22,46]. Concerning caregiver burden, alexithymic PD patients might not be able to notice the accumulating burden of their caregivers. This alexithymia-induced neglect could further increase the burden on PD caregivers. 

Many brain regions associated with alexithymia receive dopaminergic innervation as the frontal cortex and limbic structures [51]. The primary loss of dopaminergic input and secondary changes in neurotransmission might be responsible for the higher proportion of alexithymic PwP compared to the general population. In de-novo PD patients, the effect of dopaminergic treatment on alexithymia could be studied without too many confounders. Future neuroanatomical and imaging studies are needed to analyze the association of region-specific dopaminergic depletion and alexithymia.

In our study, we report a weak association of PwP alexithymia as psychological personality trait and caregiver burden. Until recently, there has been only reports in a small group of PD patients [43]. In our group of PwP with alexithymia the caregiver burden was significantly increased and caregiver QoL is significantly decreased compared to caregivers of non-alexithymic PwP. Interestingly, facet F1 “identifying feelings” and facet F2 “difficulties describing feelings” of the TAS-26 in PwP showed a significant correlation with caregiver burden. Further, we found a significant correlation of total alexithymia and facet F1 “identifying feelings” and F2 “difficulties describing feelings” with caregiver burden measured by the disease specific caregiver burden questionnaire PDCB. However, these results from linear regression analysis were not significant in the multiple regression analysis of PwP factors on caregiver burden. This might be due to the high impact of the MDS-UPDRS II score on caregiver burden. Nevertheless, by analyzing caregivers’ alexithymia, we found caregiver TAS-26 subdomain F1 “identifying feelings” and subdomain F3 “external oriented thinking” correlating significantly with caregiver burden. Interestingly, in traumatic brain disorders, Katsiferaki et al. identified alexithymia of the caregiver as a possible relevant factor for identification of relatives predisposed for caregiver burnout [52].

Until recently, no intervention in PwP has been described to decrease alexithymia. Dafsari et al. reported minor effects of subthalamic nucleus deep brain stimulation reducing alexithymia of PwP [26]. However, in healthy subjects, alexithymia has been modifiable by mindfulness interventions [53]. In general, alexithymia is a negative predictor for successful psychotherapy [54]. It is likely that this is also the case in PD patients. However, mindfulness-based interventions seem to improve alexithymia in a non-PD sample. Such interventions could help to shift more attention to feelings and emotions of the PD patient and caregiving people in their environment. This interventional technique might also lead to improved HR-QoL in these patients. However, because of deficits in the cognitive subdomain of attention in PD mindfulness-based interventions might not be as effective as in non-PD patients. The best-established intervention would be a mindfulness-based stress reduction program. This standardized intervention could be used to study the influence of mindfulness-based interventions on alexithymia in PD. In a recent study of our group, we identified caregiver mindfulness as a relevant protective factor against caregiver burden [27]. Further studies are needed to prove this assumption.

## 5. Limitations

For the recruitment of this large group of PwP and their caregivers, we decided to use a cross-sectional questionnaire-based approach. By this evaluation, we were only able to show associations and could not confirm causality. Further, because we used a questionnaire-based approach, we did not have access to extensive clinical characterizations of PwP, including the complete MDS-UPDRS [55,56]. These data could be found in a recent publication of Dafsari et al. [26]. Since we present data from a cross-sectional questionnaire-based study, we were not able to include the assessment of other neuropsychiatric symptoms in an adequate and reliable fashion. This is why we focused only on the assessment of depression in this study.

The association of cognitive deficits and alexithymia would be interesting and will be a component of future clinical trials of our group. It seems that PD patients with alexithymia might not show cognitive deficits in general compared to non-alexithymic patients [22]. However, alexithymia might be related to subdomains of cognitive tests such as the Montreal Cognitive Assessment (MoCA) test. By using these MoCA subdomains, we were able to show that executive and attentional deficits significantly impact HR-QoL [28]. Further, we would speculate that attentional deficits might also impair emotional recognition and by this promoting alexithymia.

This study was not designed to analyze the impact of different dopaminergic treatment on alexithymia [57]. In future studies, this relation has to be defined. Finally, we could not exclude a selection bias because PwP that are more interested in the alexithymia topic might have been more likely to participate in this study.

## 6. Conclusions

This study provides evidence that alexithymia of PwP is associated with decreased HR-QoL and increased caregiver burden. Since the proportion of alexithymia is markedly increased in PwP, it may be considered as an additional non-motor symptom in PD. This study suggests more robust evaluation for assessing the impact of alexithymia adjusting for possible confounders. Interventions to decrease alexithymia in PwP are needed. These treatments might include psychotherapy and mindfulness-based interventions.

## Figures and Tables

**Table 1:** Patient (*n* = 119, 45 females) and caregiver (*n* = 119, 78 females) characteristics.

	Mean ± SD	Min	Max
**PD patients**			
Age (years)	68.7 ± 10.1	40	87
Disease duration (years)	12.0 ± 7.6	1	37
Hoehn and Yahr stage	2.9 ± 1.1	1	5
MDS-UPDRS part II	19.0 ± 11.7	1	47
PDQ-8	36.9% ± 20.9%	0%	87.5%
BDI	12.6 ± 8.1	0	39
TAS-26	2.65 ± 0.6	1.1	3.9
TAS-26 F1	2.40 ± 0.83	1	4.8
TAS-26 F2	2.92 ± 0.87	1	4.8
TAS-26 F3	2.69 ± 0.65	1	4.33
**Caregivers**			
Age (years)	65.4 ± 11.0	19	87
Caregiving hours per day	5.8 ± 6.4	0	24
PDCB	37.1 ± 27.9	0	100
BDI	9.8 ± 7.3	0	36
SF-36 total	58.8 ± 17.8	5.8	85.6
TAS-26	2.33 ± 0.56	1.11	3.77
TAS-26 F1	1.99 ± 0.71	1	4
TAS-26 F2	2.5 ± 0.78	1	5
TAS-26 F3	2.59 ± 0.69	1.17	4.33

Abbreviations: BDI, Beck depression inventory; PDCB, Parkinson’s disease caregiver burden inventory; MDS-UPDRS, Movement Disorders Society unified Parkinson’s disease rating scale; PD, Parkinson’s disease; PDCB, Parkinson’s disease caregiver burden inventory; PDQ-8, Parkinson’s disease questionnaire 8; SF-36, short form 36 health survey; SD, standard deviation; TAS-26, Toronto alexithymia scale 26 (F1: difficulties identifying feelings, F2: difficulties describing feelings, and F3: external oriented thinking).

**Table 2:** Comparison of people with Parkinson’s disease with and without alexithymia.

	PwP Without Alexithymia (*n* = 83, Female 37.3%)	PwP With Alexithymia (*n* = 36, Female 38.9%)	*p*
Age	68.9 ± 9.8	68.3 ± 10.9	0.840
Disease duration	11.6 ± 6.8	12.9 ± 9.0	0.333
MDS-UPDRS II	17.4 ± 11.2	23.0 ± 11.7	0.019 *
PDQ-8	27.2 ± 16.9	48.6 ± 24.7	<0.001 **
BDI	10.4 ± 6.2	17.8 ± 9.5	<0.001 **
TAS-26 F1	2.04 ± 0.6	3.1 ± 0.66	<0.001 **
TAS-26 F2	2.6 ± 0.8	3.7 ± 0.5	<0.001 **
TAS-26 F3	2.6 ± 0.6	3.0 ± 0.6	0.001 **
Caregiver SF-36	62.2 ± 16.8	50.6 ± 17.3	0.005 *
PDCB	31.4 ± 26.8	50.9 ± 25.3	0.001 **
Caregiver BDI	8.2 ± 6.2	13.8 ± 8.2	<0.001 **
Caregiving h/d	5.2 ± 6.1	7.1 ± 7.0	0.222

*p*-value adjustment for multiple comparisons was 0.05/14. * indicate significance at *p* < 0.05. ** indicate significance level at *p* < 0.004 printed in bold. Abbreviations: BDI, Beck depression inventory; MDS-UPDRS, Movement Disorders Society unified Parkinson’s disease rating scale; PDCB, Parkinson’s disease caregiver burden inventory; PDQ-8, Parkinson’s disease questionnaire 8; PwP, people with Parkinson’s disease; TAS-26, Toronto alexithymia scale 26 (F1: difficulties identifying feelings, F2: difficulties describing feelings, and F3: external oriented thinking).

**Table 3:** Linear regression analysis of factors influencing PD patients’ quality of life measured by the disease specific assessment tool PDQ-8 (*n* = 119).

	*r*	*R* ^2^	Beta	*p*
Age	0.135	0.0182	0.135	0.153
Disease duration	0.443	0.1963	0.443	<0.001 **
MDS-UPDRS II	0.788	0.6209	0.788	<0.001 **
BDI	0.700	0.4900	0.700	<0.001 **
TAS-26	0.472	0.2228	0.472	<0.001 **
TAS-26 F1	0.557	0.3103	0.557	<0.001 **
TAS-26 F2	0.302	0.0912	0.302	0.001 **
TAS-26 F3	0.135	0.0182	0.135	0.162

Linear regression analysis of factors contributing to patients’ health-related quality of life. *p*-value adjustment for multiple comparisons was 0.05/8. ** indicate significance level at *p* < 0.006 printed in bold. Abbreviations: BDI, Beck depression inventory; MDS-UPDRS, Movement Disorders Society unified Parkinson’s disease rating scale; PD, Parkinson’s disease; PDQ-8, Parkinson’s disease questionnaire 8; TAS-26, Toronto alexithymia scale 26 (F1: difficulties identifying feelings, F2: difficulties describing feelings, and F3: external oriented thinking).

**Table 4:** Multiple regression analysis of factors influencing quality of life of PD patients measured by the disease specific assessment tool PDQ-8 (*n* = 104).

	B (95% CI)	*R* ^2^	Beta	*t*	*p*
(Constant)	6.690 (3.989; 9.392)			4.914	<0.001 *
Disease duration	0.075 (−0.034; 0.184)	0.288	0.083	1.360	0.177
MDS-UPDRS II	0.314 (0.234; 0.395)	0.729	0.531	7.749	<0.001 *
BDI	0.233 (0.110; 0.356)	0.531	0.282	3.756	<0.001 *
TAS-26 F1	1.631 (0.417; 2.845)	0.445	0.202	2.666	0.009 *
TAS-26 F2	−0.405 (−1.365; 0.554)	−226	−0.051	−0.839	0.404

* Indicates significant correlations at *p* < 0.05 printed in bold. Abbreviations: BDI, Beck depression inventory; MDS-UPDRS, Movement Disorders Society unified Parkinson’s disease rating scale; PD, Parkinson’s disease; PDQ-8, Parkinson’s disease questionnaire 8; TAS-26, Toronto alexithymia scale 26 (F1: difficulties identifying feelings, F2: difficulties describing feelings, and F3: external oriented thinking).

**Table 5:** Linear regression analysis of factors influencing caregiver burden measured by the disease specific assessment tool PDCB (*n* = 119).

	*r*	*R* ^2^	Beta	*p*
PDQ-8	0.563	0.317	0.563	<0.001 **
MDS-UPDRS part II	0.610	0.372	0.610	<0.001 **
Patient BDI	0.432	0.187	0.432	<0.001 **
Patient TAS-26	0.373	0.139	0.373	<0.001 **
Patient TAS-26 F1	0.352	0.124	0.352	<0.001 **
Patient TAS-26 F2	0.333	0.111	0.334	0.001 **
Patient TAS-26 F3	0.145	0.021	0.147	0.138
Caregiving hours per day	0.475	0.225	0.475	<0.001 **
Caregiver SF-36	0.541	0.292	−0.541	<0.001 **
Caregiver SF-36 Physical Health	0.446	0.199	−0.446	<0.001 **
Caregiver SF-36 Mental Health	0.584	0.341	−0.584	<0.001 **
Caregiver BDI	0.513	0.263	0.513	<0.001 **
Caregiver TAS-26	0.324	0.105	0.324	0.001 **
Caregiver TAS-26 F1	0.255	0.065	0.256	0.008 *
Caregiver TAS-26 F2	0.176	0.031	0.0177	0.069
Caregiver TAS-26 F3	0.281	0.079	0.280	0.004 *

Linear regression analysis of patient and caregiver factors contributing to caregiver burden. *p*-value adjustment for multiple comparisons was 0.05/14. * indicates significant correlations at *p* < 0.05; ** indicate significance level at *p* < 0.004 printed in bold. Abbreviations: BDI, Beck depression inventory; MDS-UPDRS, Movement Disorders Society unified Parkinson’s disease rating scale; PDCB, Parkinson’s disease caregiver burden inventory; PDQ-8, Parkinson’s disease questionnaire 8; SF-36, short form 36 health survey; TAS-26 Toronto alexithymia scale 26 (F1: difficulties identifying feelings, F2: difficulties describing feelings, and F3: external oriented thinking).

**Table 6:** Multiple regression analysis of patient factors influencing caregiver burden measured by the disease specific assessment tool PDCB (*n* = 78).

	B (95% CI)	*R* ^2^	Beta	*t*	*p*
(Constant)	−13.392 (−35.937; 9.153)			−1.180	0.241
PwP disease duration	−0.002 (−0.702; 0.698)	0.000	0.000	−0.005	0.996
PDQ-8	0.559 (−0.706; 1.825)	0.365	0.133	0.878	0.382
MDS-UPDRS part II	1.030 (−0.388; 1.673)	0.644	0.415	3.185	0.002 *
PwP BDI	0.213 (−1.318; 0.820)	0.249	0.062	0.532	0.598
PwP TAS-26	6.827 (−2.255; 15.910)	0.378	0.143	1.493	0.139

* indicates significant correlations at *p* < 0.05 printed in bold. Abbreviations: BDI, Beck depression inventory; MDS-UPDRS, Movement Disorders Society unified Parkinson’s disease rating scale; PDCB, Parkinson’s disease caregiver burden inventory; PDQ-8, Parkinson’s disease questionnaire 8; PwP, people with Parkinson’s disease; SF-36, short form 36 health survey; SD, standard deviation; TAS-26 Toronto alexithymia scale 26 (F1: difficulties identifying feelings, F2: difficulties describing feelings, and F3: external oriented thinking).
